# Mechanisms of bariatric surgery for weight loss and diabetes remission

**DOI:** 10.1111/1753-0407.13443

**Published:** 2023-07-13

**Authors:** Mengsha Yin, Yao Wang, Mingyue Han, Ruishuang Liang, Shanshan Li, Guixia Wang, Xiaokun Gang

**Affiliations:** ^1^ Department of Endocrinology and Metabolism The First Hospital of Jilin University Changchun China; ^2^ Department of Orthopedics The Second Hospital Jilin University Changchun China

**Keywords:** bariatric surgery, diabetes, intestinal hormones, obesity, mechanism, 减重手术, 糖尿病, 肠道激素, 肥胖, 机制

## Abstract

Obesity and type 2 diabetes(T2D) lead to defects in intestinal hormones secretion, abnormalities in the composition of bile acids (BAs), increased systemic and adipose tissue inflammation, defects of branched‐chain amino acids (BCAAs) catabolism, and dysbiosis of gut microbiota. Bariatric surgery (BS) has been shown to be highly effective in the treatment of obesity and T2D, which allows us to view BS not simply as weight‐loss surgery but as a means of alleviating obesity and its comorbidities, especially T2D. In recent years, accumulating studies have focused on the mechanisms of BS to find out which metabolic parameters are affected by BS through which pathways, such as which hormones and inflammatory processes are altered. The literatures are saturated with the role of intestinal hormones and the gut‐brain axis formed by their interaction with neural networks in the remission of obesity and T2D following BS. In addition, BAs, gut microbiota and other factors are also involved in these benefits after BS. The interaction of these factors makes the mechanisms of metabolic improvement induced by BS more complicated. To date, we do not fully understand the exact mechanisms of the metabolic alterations induced by BS and its impact on the disease process of T2D itself. This review summarizes the changes of intestinal hormones, BAs, BCAAs, gut microbiota, signaling proteins, growth differentiation factor 15, exosomes, adipose tissue, brain function, and food preferences after BS, so as to fully understand the actual working mechanisms of BS and provide nonsurgical therapeutic strategies for obesity and T2D.

## Graphical abstract

**Highlights**

- Controversial changes in intestinal hormones after bariatric surgery (BS) were summarized.
- The changes of signaling proteins involved in insulin resistance and the newly proposed changes in growth differentiation factor 15, exosomes, and extracellular vesicles after BS were elucidated.
- The interactions between bile acids and gut microbiota and the gut–brain axis play an indispensable role in BS.

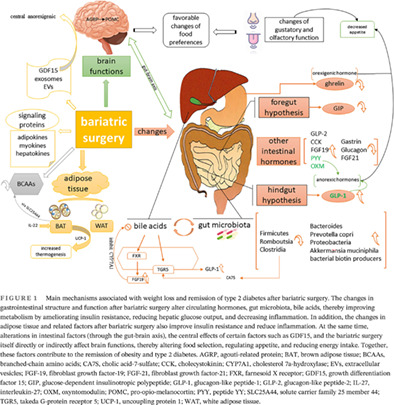

## 摘要

肥胖和2型糖尿病(T2D)导致肠道激素分泌缺陷、胆汁酸(BA)合成异常、脂肪组织炎症增加、支链氨基酸分解代谢异常和肠道菌群失调。减重手术(BS)已被证明在治疗肥胖和2型糖尿病方面非常有效, 这使得我们不能将BS简单地视为减重手术, 而是一种减轻肥胖及其合并症, 尤其是2型糖尿病的手段。近年来, 越来越多的研究关注BS的发病机制, 试图找出BS通过哪些途径影响代谢参数, 如哪些激素和炎症过程发生改变。目前已有大量文献证明肠道激素及其与神经网络相互作用形成的脑‐肠轴在BS后肥胖和T2D缓解中的作用。此外, BS后的益处还与BAs、肠道菌群等因素有关。这些因素的相互作用使得BS引起代谢改善的机制更加复杂。迄今为止, 我们还没有完全了解BS诱导的代谢改变的确切机制及其对T2D本身疾病过程的影响。本文综述了BS后肠道激素、BAs、BCAAs、肠道菌群、信号蛋白、生长分化因子15、外泌体、脂肪组织、脑功能、食物偏好等方面的变化, 以期全面了解BS的实际作用机制, 为肥胖和T2D的非手术治疗提供策略。

## 1 INTRODUCTION

The Global Burden of Diseases, Injuries, and Risk Factors Study (GBD) 2019 revealed that the prevalence of all metabolic diseases increased from 2000 to 2019.^1^ The latest national prevalence estimates for 2015–2019 were 34.3% for overweight and 16.4% for obesity in adults (≥18 years) according to Chinese criteria.^2^ According to GBD data, the absolute burden of obesity is highest and the number of deaths is highest, with a total of 5.0 million deaths in 2019, in addition to an estimated 43.8 million type 2 diabetes (T2D) patients worldwide in 2019, with 1.4 million deaths among these patients.^1^ Moreover, the mortality rates of obesity and T2D did not decrease over time.^1^ Obesity is a complex disease with multiple etiologies, with its own disabling capabilities and comorbidities.^3^, ^4^ Studies have shown that obesity is the strongest risk factor for the development of diabetes^5^ and is also associated with an increased risk of cardiovascular diseases, liver diseases, and some kinds of cancers.^6^, ^7^ Statistically, the largest proportion of metabolic disease‐related mortality was contributed by obesity.^1^ Basic treatments for obesity include low‐calorie low‐fat diets, increased physical activity, and strategies that contribute to the modifications in lifestyle. Antiobesity drugs contribute to weight loss and further improve the health risks.^7^ However, existing antiobesity drugs, including sympathomimetics, GABA_A_ receptor activators, pancreatic lipase inhibitors, a serotonin 2C receptor agonist, dopamine–norepinephrine reuptake inhibitor, opioid antagonist, and glucagon‐like peptide‐1 (GLP‐1) receptor agonists, are not as effective as desired, and they have side effects.^8^ For example, topiramate may cause increased pulse and blood pressure in certain patients and increase the risk of oral clefts in infants when taken by pregnant women; liraglutide has obvious gastrointestinal side effects, such as nausea, vomiting, diarrhea, and an increased risk of pancreatitis.^8^ Moreover, short‐term weight control is easily achieved by the means described, but it is prone to weight regain.^9^, ^10^ There are certain advantages in maintaining long‐term weight loss for bariatric surgery (BS) in severely obese patients.^7^, ^11^ BS, also known as metabolic surgery, has been around since the 1950s. Over the years, there have been significant changes in access and types of BS, most notably the almost exclusive adoption of laparoscopic techniques in the specialty. Other significant changes were related to the techniques: the use of vertical banded gastroplasty (VBG) declined in the late 1990s, laparoscopic adjustable gastric banding (AGB) emerged around 2012, and thereafter the utilization of biliopancreatic diversion (BPD) remained at its lowest level, maintaining a relatively large proportion of Roux‐en‐Y gastric bypass (RYGB) for a long time; as well as the utilization of laparoscopic sleeve gastrectomy (SG) has increased rapidly in recent years.^12^ For decades, BS has proven successful in achieving meaningful and sustainable weight loss in a large number of patients undergoing surgery.^13^ In addition, BS significantly improves comorbidities as well as reduces overall mortality by 25%–50% during long‐term follow‐up.^7^ Although the benefits of BS are clear, the means by which it is achieved remain to be elucidated. Different surgical methods result in different changes in the structure of gastrointestinal tract, based on the similarities and differences in weight loss and metabolic improvement in these procedures. Clinical and animal studies have focused on the changes of intestinal hormones, gut microbiota, bile acids (BAs), and circulating immune and cytokine production following BS. At present, there are a number of controversial opinions about how these various mechanisms work and how they intersect and overlap. This review is intended to elucidate the mechanisms of benefit of BS for weight loss and diabetes remission form various aspects and thus to provide ideas for unlocking other noninvasive treatment strategies against obesity and related comorbidities that were previously unknown.

## 2 MECHANISMS OF BARIATRIC SURGERY

### 2.1 Weight loss

Metabolic changes were frequently observed soon after BS and BS is more effective than dietary control in improving diabetes, even with equivalent weight loss. For example, with the same weight loss, postprandial glucose levels decreased more and the GLP‐1 levels increased more at 1 month after gastric bypass surgery (GBP) compared with a low‐caloric diet,^14^ indicating that mechanisms may be partially independent of weight loss.^15^, ^16^

### 2.2 Dietary restriction

Dietary restriction after BS appears to play a major role in weight loss. In addition to a smaller gastric pouch, reduced appetite such as the decrease in orexigenic hormones after BS and the increase in anorexic hormones contributes to a further decrease in energy intake. These hormonal changes echo the changes in brain function projected by functional magnetic resonance imaging (fMRI) described below. Specifically, patients undergoing RYGB have increased postprandial plasma GLP‐1,^17^ peptide YY (PYY),^18^, ^19^ and oxyntomodulin (OXM),^20^ which are beneficial to enhancing satiety and thus lead to a reduction in energy intake.^21^ Ghrelin is a peptide of 28 amino acids originally discovered in 1999.^22^ It acts as an orexigenic hormone and is mainly secreted by the stomach.^22^, ^23^ Ghrelin has diverse biological functions in regulating energy homeostasis, including the abilities to communicate with the hypothalamus about current peripheral nutritional status and to compensate for energy.^24^ It is also associated with increased plasma levels of insulin, glucagon, and leptin.^25^ Studies have shown that ghrelin impairs carbohydrate and lipid metabolism in obese patients and BS such as SG,^18^ AGB,^26^ and GBP^27^, ^28^ are associated with significantly suppressed ghrelin levels. However, there have been some controversial findings regarding ghrelin level after BS, such as studies showing that ghrelin concentrations increased to 40% above baseline levels in patients receiving BPD‐RYGB who completed 1 year of follow‐up^19^ and plasma ghrelin increased at 1 year in patients undergoing AGB.^29^ Another study reported that there was no significant change in fasting ghrelin levels from baseline at 1 year after RYGB, nor was there a significant reduction in ghrelin levels after the test meal.^18^ The discrepancies in ghrelin levels after BS can be attributed to differences in surgical techniques among centers, including the remaining size of the gastric pouch, the handling of the vagus nerve, the length of Roux‐limp, and the timing of samplings. However, orexigenic hormones were significantly attenuate after RYGB but not after weight loss with the equivalent caloric restriction^30^ and RYGB results in improved metabolic flexibility, such as more complete β‐oxidation of fatty acids and greater handling of glucose and amino acids, compared with the equivalent dietary restriction,^31^ indicated that mechanisms other than energy restriction cannot be excluded.

### 2.3 The hindgut hypothesis and the foregut hypothesis

Two major hypotheses have been proposed to explain the effects of BS on T2D: the hindgut hypothesis^32^, ^33^ and the foregut hypothesis.^33^, ^34^, ^35^ The former points out that remission of T2D results from faster delivery of nutrients to the distal small intestine,^36^ where L‐cells are more densely distributed^37^ and thus enhances the release of GLP‐1.^38^, ^39^, ^40^ GLP‐1 stimulates not only insulin secretion but also proinsulin gene transcription and insulin biosynthesis and inhibit glucagon secretion,^41^, ^42^, ^43^ a physiological marker of improved glucose metabolism. Some studies have shown that GLP‐1 is dispensable for the metabolic effects after BS; for example, Albaugh et al found that the glucose‐regulating effect of bile diversion to the ileum was abolished in whole‐body GLP‐1 receptor (GLP‐1R) deficient mice.^44^ The effect of GLP‐1R agonists such as semaglutide and liraglutide on weight loss is comparable to that of BS, which seems to confirm the important role of GLP‐1. However, infusion of exendin‐(9–39), a GLP‐1R antagonist,^45^ resulted in a slight deterioration of postprandial plasma glucose in RYGB subjects, suggesting that the resolution of T2D after RYGB may be explained by mechanisms other than enhanced GLP‐1 action.^46^ Similarly, GLP‐1 played a limited role in short‐term glycemic improvement after RYGB compared with intensive lifestyle management.^47^ The metabolic benefits induced by SG^48^ and RYGB^49^ could overcome the defects in glucose regulation due to the lack of GLP‐1 signaling in β‐cells in obese mice, further demonstrating that the hindgut hypothesis may not fully explain the benefits of BS. The foregut hypothesis, on the other hand, suggests that exclusion of the proximal small intestine from nutrient transit would reduce or suppress the secretion of anti‐incretin hormones that promote insulin resistance, thereby improving glycemic control.^50^ Glucose‐dependent insulinotropic polypeptide (GIP) is an intestinal hormone that is secreted by K cells distributed in the proximal small intestine^37^ and promotes the storage of both glucose and fat.^51^ GIP exhibits incretin activity and increases insulin secretion in hyperglycemia. Increased GIP signaling plays an important role in adipose tissue inflammation and insulin resistance in obese mice.^52^ Studies using GIP receptor knockout mice suggest that GIP action is important in fat deposition and that inhibition of GIP signaling may be a target for obesity.^51^, ^53^, ^54^ The metabolic benefits of bypass surgery are mediated in part by surgical removal of GIP‐secreting K cells in the proximal small intestine^55^, ^56^ and emerging evidences have also shown that a rapid reduction in GIP levels following RYGB^57^ and BPD.^58^, ^59^ Treatment of obese mice with GIP receptor (GIPR) antagonist leads to rapid improvement in β‐cell function and glucose tolerance through alleviation of insulin resistance,^60^ but it remains to be investigated whether the effects of blocking GIP in humans are similar to those observed in mice. Some studies showed that bypassing of the jejunum or a short segment of the proximal gut is beneficial with respect to insulin‐mediated glucose disposal in obese patients, independent of effects on body weight, food intake, or hindgut nutrient delivery.^34^, ^35^, ^61^ Interestingly, the longer the portion of the jejunal bypass during metabolic surgery was associated with greater improvement in insulin sensitivity.^61^ Similarly, remission rates of T2D were higher after BPD than after RYGB in clinical trials because in RYGB, only the duodenum is bypassed, whereas in BPD, the duodenum and part of jejunum are excluded from food transport.^33^, ^62^, ^63^ It was also found that anatomical alterations in the proximal small intestine may reduce factors associated with negative effects on insulin sensitivity, contributing to the control of diabetes after GBP in rats.^64^ Interestingly, the superior efficacy of GIPR‐ GLP‐1R coagonist tirzepatide^65^ in T2D has drawn attention to the role of GIP in metabolism in recent years. It is possible that both the increase and decrease of GIPR signaling reduce body weight, and further studies are needed to investigate the role of GIP in humans. However, no significant differences were found between RYGB and SG in improving glucose homeostasis; increasing insulin, GLP‐1, and PYY levels^66^; and promoting weight loss at long‐term follow‐up,^67^, ^68^, ^69^, ^70^ which do not support the foregut hypothesis. Moreover, neither the changes in GLP‐1 plasma levels nor the changes in GIP explained the normalization of insulin sensitivity, this fact may indicate the presence of other intestinal factors.

### 2.4 Changes in other intestinal hormones

Some studies showed that insulin sensitivity increased more with oral glucose than with intravenous glucose after BS, suggesting the importance of intestinal hormones. The roles of ghrelin, GLP‐1, and GIP have been described earlier, and the roles of other hormonal changes in BS should be further explored. In the mid‐ to late 1980s, it was recognized that glucagon‐like peptide‐2 (GLP‐2) is specifically processed from preproglucagon in the gut^71^, ^72^ and is cosecreted with GLP‐1.^73^ Similar to GLP‐1, GLP‐2 is also increased after RYGB to promote the proliferation of mucosal crypt cells^74^ through insulin‐like growth factor‐1 receptor in intestinal epithelium,^75^ which may be beneficial for the restoration of intestinal absorptive surface area, thereby limiting malabsorption and promoting long‐term weight loss in rodents and humans.^76^ GLP‐2 was significantly elevated after RYGB and correlated with satiety in clinical trials,^77^ as was GLP‐2 observed after SG.^78^ However, the loss of GLP‐2 receptor (GLP‐2R) did not attenuate the extent of weight loss and improved glycemic control after SG in mice, demonstrating that GLP‐2R signaling is dispensable for the metabolic benefits generated after SG.^79^

PYY3‐36 reduces food intake in normal‐weight subjects by modulating hypothalamic appetizing circuits; however, obese subjects have been found to have lower endogenous PYY levels and are not resistant to the anorectic effects of PYY.^80^ Meanwhile, attenuated postprandial PYY secretion was observed in the early stages of T2D development.^81^ Many studies have found that PYY levels increase after BS such as RYGB^18^, ^82^, ^83^, ^84^ or SG.^18^, ^66^ Ramracheya et al demonstrated that diabetic rats undergoing RYGB rely on PYY to restore impaired glucose‐mediated insulin and glucagon secretion.^85^ Tests of human islet function before and after BS, in the presence or absence of PYY, demonstrated that PYY plays a key role in the resolution of T2D in BS.^86^ However, a prospective study of severely obese individuals (25 nondiabetic and 10 diabetic) who underwent RYGB found that increased PYY levels were associated with sustained weight loss after surgery. However, there was no significant correlation between PYY and glucose tolerance in either group.^82^ Regardless, the beneficial metabolic effects of BS are mediated through changes in PYY levels remain to be proven.

Gastrin and cholecystokinin (CCK) are known homologous hormone systems, which not only regulate gastric acid secretion and the growth of gastric and pancreatic cells but also participate in the development and secretion of islet cells.^87^ It has been shown that resection of distal gastric mucosa significantly reduced body weight and improved glycemic control in rats, inferring that the decrease in gastrin level caused by gastric mucosa exclusion in RYGB may be the key for weight loss and T2D remission.^88^ Due to structural changes in the stomach caused by surgery, gastrin level decreased after RYGB,^89^ whereas others have shown that gastrin level was unchanged after AGB^90^ and increased significantly after SG in rodents.^91^ As for the CCK, Rhee et al found that the distribution of enteroendocrine cells underwent a lot of alterations after RYGB in obese patients with T2D, including an increased density of CCK‐positive cells.^92^ It has been found that infusion of nutrients into the bypassed jejunum after the jejunal bypass stimulated CCK secretion and pancreatic growth in rats.^93^ However, other studies have shown that CCK response to meals is not changed after RYGB^57^ and VBG compared with presurgery, suggesting that CCK does not mediate the endocrine satiety effect of BS.^94^ These controversial changes in gastrin and CCK after different bariatric surgeries indicate the need for further studies exploring their association with BS.

OXM, a postprandial peptide hormone released from the gut, activates both GLP‐1R and glucagon receptor (GCGR) to induce weight loss,^95^ inhibit food intake^96^ and regulate energy expenditure.^97^ Some studies have shown that its analogue eliminated obesity and diabetes in mice.^98^ Combined injection of OXM, GLP‐1, and PYY improved body weight and hyperglycemia compared with placebo in humans.^99^, ^100^ Perakakis et al found that postprandial OXM levels increased most strongly at 3 months after SG and were associated with the degree of weight loss, which serve as predictors of weight loss, presumably by regulating satiety.^101^ Similarly, OXM levels were significantly increased 1 month after RYGB compared with the diet‐induced equivalent weight loss and were significantly correlated with GLP‐1 and PYY.^20^ OXM is derived from the proglucagon gene and has structural similarity to glucagon. Glucagon is known to be released in the fasting state and increases blood glucose levels by promoting glycogenolysis and gluconeogenesis. There are few studies on glucagon in BS, and the present studies have shown that glucagon levels were decreased after SG^102^ and RYGB.^103^

Fibroblast growth factors 19 (FGF19) and 21 (FGF21) are secreted by the intestine and liver and have emerged as key regulators of energy metabolism. The biological effects of FGF21 include weight loss by reducing food intake and increasing energy expenditure, as well as lowering plasma glucose by increasing insulin sensitivity. However, FGF21 levels are elevated in obese patients and are further increased in obese patients with T2D, so obesity is proposed to be a FGF21‐resistant state.^104^ Instead, circulating serum FGF19 concentrations are significantly decreased in obese and T2D patients. Many studies have yielded controversial results regarding the changes in FGF19 and FGF21 after BS. It has been shown that FGF21 levels decreased after SG^105^ and GBP^106^ induced weight loss, whereas they remained unchanged after RYGB.^105^ Moreover, other studies have shown that FGF21 concentrations elevated after RYGB in 16 obese patients.^107^ The reason why FGF21 levels are unchanged or even increased after RYGB may be related to the changes in intestinal structure that cause the rapid delivery of nutrients to the small intestine and glucose delivery to the liver or reverse FGF21‐resistance state. FGF19 inhibits gluconeogenesis and stimulates glycogen synthesis but does not increase lipogenesis. FGF19 concentrations increased after weight loss induced by SG, RYGB, and AGB^108^ in obese patients, as well as after GBP^106^ in obese patients with T2D. The role of FGF19 in BS is elaborated in a later section.

It has been proposed that circulating follistatin and its homologous protein, follistatin‐like 3 play an important role in glucose homeostasis and they were decreased after RYGB and SG, and were correlated with the changes of blood glucose, insulin, and glycosylated hemoglobin.^109^ Circulating succinate was significantly reduced after BS and had predictive value for T2D remission proposed by Victoria et al, obese patients with T2D who with different baseline succinate levels had different responses to the type of surgery and different T2D remission rates.^110^

There are also many hormones such as insulin, secretin, pancreatic polypeptide, obestatin, and so on that play certain roles in weight loss and metabolic improvement after BS, which are not discussed in this review.

### 2.5 Changes in signaling proteins (adipokines, myokines, hepatokines), GDF15, exosomes, and adipose tissue

Altered adipokines levels may contribute to metabolic dysfunction in obesity. The extent of adipokines changes after BS and their impact on metabolic improvements have been explored in several studies. Adiponectin levels increase^111^, ^112^, ^113^ and leptin levels decrease after BS, and surgery shifts the adipokines profiles of obese patients toward lean controls.^114^ Specifically, in a prospective controlled Swedish Obese Subjects Study, adiponectin levels were compared between 1570 subjects undergoing BS and 1729 controls receiving usual care. The results suggest that the magnitude of weight loss after BS paralleled a significant increase in circulating high molecular weight adiponectin.^115^, ^116^ Patients with T2D remission after BS have higher levels of adiponectin and lower high‐sensitivity C‐reactive protein than those without remission, and elevated adiponectin is associated with enhanced β‐cells function, greater fat loss, and lower triglyceride levels,^117^ which indicates that inflammation and insulin resistance may be reduced. Leptin is an anorexigenic hormone that is secreted by white adipose tissue, and despite the anorectic effect of plasma leptin, it is correlated with body fat content, suggesting that obesity is associated with a state of leptin resistance.^118^, ^119^ Moreover, leptin resistance may account for the decreased GLP‐1 levels in obese individuals.^120^ It is reported that leptin levels decreased at 1 year after RYGB and AGB.^29^, ^121^

The activity of brown adipose tissue (BAT) protects against obesity and T2D.^122^ Thermogenesis in BAT (both brown and beige adipocytes) plays an important role in combating the development of metabolic disorders.^123^, ^124^, ^125^ Recently, Qian Wang et al found that interleukin‐27 (IL‐27) directly acted on BAT, stimulating uncoupling protein 1 (UCP‐1) production to increase thermogenesis, protect against obesity and ameliorate insulin resistance.^126^ The serum IL‐27 levels were significantly reduced in obese individuals with T2D and were restored after RYGB,^127^ indicating that BS may improves the regulation of BAT metabolism by restoring the levels of certain factors. Obesity and T2D are associated with low‐grade chronic inflammation of white adipose tissue (WAT), increased proinflammatory cytokines and local infiltration of immune cells lead to insulin resistance in obese patients.^122^ Genes encoding inflammation‐related proteins in WAT continued to decline 2 and 5 years after RYGB in 38 obese patients, indicated that the metabolic effects of BS may be related in part to altered gene expression in WAT.^128^ However, another study showed that elevated leukocyte infiltration and unchanged proinflammatory cytokine mRNA expression in adipose tissue at 1 month or 6 to 12 months after BS in 17 obese patients, reflect that neither short‐term nor long‐term metabolic improvement after BS significantly reduces inflammatory markers of adipose tissue. This result reveals that reduction in adipose tissue inflammation did not contribute to the metabolic benefits of BS.^129^ The different results may be due to different populations, surgical centers, and follow‐up time. In addition, both myokines and hepatokines are associated with insulin resistance in obesity. Metabolic changes induced by BS appear to be related to reduction in myokines.^130^ There is limited research on the role of hepatokines, including insulin‐like growth factor binding protein 2 (IGFBP2), adropin, and sex hormone binding globulin, after BS. Among them, IGFBP2 is significantly increased after RYGB in humans, rats, and mice, and deletion of IGFBP2 impairs weight loss and early improvement in insulin sensitivity induced by surgery, suggesting a potential role of circulating IGFBP2 in BS.^131^ The remaining factors require further research. Growth differentiation factor 15 (GDF15), a cytokine that reduces food intake by exerting central anorexigenic effects, has anti‐inflammatory effects and increases insulin sensitivity, which may improve clinical outcomes in patients with obesity and T2D.^132^ Studies have shown that the levels of GDF15 increased after SG in mice^133^ and humans,^134^ as well as after RYGB,^133^, ^135^ but lack of GDF15 signaling did not alter food intake or body weight after SG, indicating that GDF15 may not be essential for the potent effects of SG,^136^ further studies are needed to explore the role of GDF15 in BS. Recent studies have highlighted the role of exosomes in mediating the crosstalk between liver, skeletal muscle and adipose tissue during the development of insulin resistance.^137^ Exosomal microRNAs (miRNAs) have emerged as potential biomarkers of obesity. Exosomes derived from obese adipose contain dysregulated miRNAs associated with insulin signaling compared to lean controls, but circulating exosomes are modified following BS and associated with improved insulin resistance.^138^, ^139^ Extracellular vesicles (EVs) are crucial modes of intercellular communication, modulating multiple biological processes by carrying hormones, nucleic acids, and signaling molecules.^140^ Obesity interferes with the function of human adipose mesenchymal stem/stromal cells (ASCs), thereby altering the size and miRNAs cargo of ASCs‐derived EVs and reducing their ability to repair damaged cells.^141^ In mice experiments, the composition of intestinal EVs altered substantially after VSG and may regulate various signaling pathways.^140^ Extracellular miRNAs regulate cellular metabolism by mediating intercellular communication.^142^ These miRNAs are partially found in small vesicles/exosomes, and circulating miRNAs have been linked to metabolic disorders. A longitudinal study in humans revealed that 42 circulating miRNAs were differentially expressed between 6 and 12 months after RYGB. Among these, circulating miR‐15a, miR‐22, and miR‐192 were increased in nine obese individuals with T2D and positively correlated with disease severity, whereas they decreased after RYGB.^143^ Circulating levels of miR‐92a were positively associated with body mass index (BMI) and impaired glucose metabolism, but decreased at 6 months following BS.^144^ Thus, alterations in circulating miRNAs may partly explain the improved metabolic function after BS. However, how BS leads to changes in circulating miRNAs and how these miRNAs participate in regulating systemic metabolism require further investigation.

### 2.6 Changes in the concentrations and compositions of bile acids

Increasing evidence suggests that the balance of BAs synthesis pathways (between the classical pathway and the alternative pathway) may be a therapeutic target for metabolic disorders. BAs are important metabolic regulator acting through the Takeda G‐protein receptor 5 (TGR5) and the Farnesoid X receptor (FXR). BS may improve metabolism by affecting the concentrations and compositions of BAs. For example, SG improved glucose homeostasis by increasing the levels of circulating BAs and the signals of BAs via TGR5. Experiments in mice have shown that relative to TGR5 ^+/+^ mice, the weight‐independent improvements in fasting plasma glucose, glucose tolerance, and hepatic insulin signaling following SG were attenuated in TGR5 ^−/−^ mice.^145^, ^146^ Similarly, notoginsenoside Ft1 was identified as a TGR5 agonist in vitro, which promoted fat browning in adipose tissue, increased lipolysis, and induced GLP‐1 secretion in obese mice, and these effects were not observed in TGR5 ^−/−^ mice.^147^ The signals of BAs also act through another receptor: FXR. Ryan et al found that in the absence of FXR, the ability of SG to reduce body weight and improve glucose tolerance was greatly reduced.^148^ Lili Ding et al also demonstrated that FXR knockout mice fed a high‐fat diet were resistant to the beneficial metabolic effects of SG.^149^ These findings suggest that changes in the circulating BAs pool after BS play an important role in metabolic improvement through TGR5 and FXR. Treatment with the TGR5/FXR coagonist INT‐767 resulted in weight loss and improved glucose tolerance in obese mice.^150^ However, the role of FXR in obesity and T2D remains controversial, with conflicting results reported in different studies. For instance, gut‐restricted FXR agonist fexaramine (Fex) was shown to enhance thermogenesis and browning of WAT, reducing obesity and insulin resistance in mice.^151^ In contrast, tempol reduced obesity in mice by increasing intestinal tauro‐β‐muricholic acid, a FXR nuclear receptor antagonist.^152^ This discrepancy led researchers to focus on FGF19, a downstream target gene of FXR. In ileal cells, BAs activate FXR and its downstream target, FGF19. FGF19 enters the liver through the portal venous circulation to bind to its receptor and represses BAs synthesis by inhibiting CYP7A1 (cholesterol 7a‐hydroxylase, a rate‐limiting enzyme for BAs synthesis).^153^ In a study of 115 patients with T2D who underwent RYGB, those who experienced complete remission of T2D after surgery were found to have higher levels of FGF19, this suggested an important role for the FGF19‐CYP7A1‐BAs pathway in the etiology and remission of T2D after RYGB.^154^ FGF19 variant M70 (NGM282) has been shown to reduce liver fat content in humans. As mentioned previously, FGF19 levels are increased after BS, suggesting that it may be a potential target for mediating the beneficial effects of BS, but the specific pathways by which it is mediated remain unclear. Studies have shown that the changes in compositions of BAs after BS such as the increase of lithocholic acid in the portal vein of mice following SG induced the production of cholic acid‐7‐sulfate (CA7S) by activating the vitamin D receptor, which acted on the TGR5 to induce GLP‐1 secretion,^155^ thereby improving metabolism.^156^ It has been hypothesized that the increased delivery of BAs to distal L‐cells may contribute to the increase of gut peptide secretion after BS, but research^157^ showed that GLP‐1 and PYY increased rapidly after surgery, whereas BAs significantly increased at 1 year after BS, indicating that BAs do not seem to be the key regulator of the early postoperative increase of gut peptide. In addition, the changes of intestinal BAs after BS are controversial for the improvement of metabolism. For example, restoration of small intestinal BAs levels partially blocked the beneficial effects of SG in mice,^149^ whereas some studies have found that in obese rats, the alterations in the gut microbiome caused by RYGB result in an increase in luminal and systemic pools of taurine‐conjugated bile acids, which induce signaling through FXR and TGR5 to improve metabolism.^158^ The difference may be related to the different surgical procedures and species of BAs.

### 2.7 Changes of branched‐chain amino acids

The levels of circulating BCAAs (leucine, isoleucine and valine) were significantly elevated in individuals with T2D or obesity with insulin resistance.^159^, ^160^, ^161^ BAT utilizes BCAAs in mitochondria for thermogenesis and controls BCAAs clearance through solute carrier family 25 member 44, thereby improving metabolism and in turn, defects of BCAAs catabolism in BAT were associated with obesity in mice.^159^, ^162^ BCAAs decreased significantly after RYGB, BPD, and SG^163^, ^164^ and this change lasted up to 12 months after RYGB and SG.^165^ In addition, some studies have shown that although both BS (such as GBP and RYGB) and calorie restriction resulted in significant weight loss, the former induced a reduction in BCAAs levels but the latter did not, suggesting a BS‐dependent mechanism for BCAAs reduction.^166^, ^167^ The effects of sodium phenylbutyrate (NaPB) on metabolic health in 16 patients with T2D were evaluated in a randomized, placebo‐controlled trial. The results showed that NaPB resulted in an 8% reduction in BCAAs levels at 2 weeks, a 27% improvement in peripheral glucose disposal, and an increase in muscle mitochondrial oxidative capacity. These findings suggest that NaPB, a promoter of BCAAs catabolism, may be a promising treatment approach for T2D.^168^ However, Kramer et al indicated that increased circulating BCAAs did not attenuate the benefits of SG in mice, suggesting that reductions in BCAAs were not essential for sustained weight loss and improved glucose tolerance following SG.^169^ Whether the reductions of BCAAs after BS play an independent role remains to be demonstrated.

### 2.8 Metabolic alterations in the gut microbiota

Emerging evidence has shown that obese individuals have abnormal gut microbiota^170^, ^171^, ^172^, ^173^ and human microbiome influences insulin sensitivity.^160^ Over the years, it identified the alterations of major gut microbiota in severe obesity, which include reduced microbial gene richness (MGR) and associated functional pathways related with metabolic deterioration. AGB and RYGB increased MGR at 1 year after surgery, improved metabolism and inflammation in 61 severe obese subjects, and was associated with changes in gut microbiota.^174^ Specifically, SG resulted in an increase in the abundance of *Bacteroides thetaiotaomicron* in mice^156^ and a decrease in serum glutamate concentration, which partially reversed obesity‐related microbial and metabolic alterations.^175^ In addition, obese mice gavaged with *Bacteroidetes spp*. exhibited attenuated body‐weight gain^175^ and improved BCAAs catabolism in BAT.^162^ These results identify the links between obesity, intestinal microbiota, and circulating amino acids, suggesting that it is possible to intervene in obese individuals by targeting the Bacteroides probiotics. Cecal *Prevotella copri* was significantly enriched in Goto‐Kakizaki rats with spontaneous T2D after SG, and glucose homeostasis was improved through enhanced bile acid metabolism and FXR signaling.^176^ However, *Bacteroides vulgatus* and *Prevotella copri* are mainly involved in the biosynthesis of BCAAs, thereby increasing the levels of BCAAs and inducing insulin resistance in mice.^160^ A similar paradox is that BCAAs supplementation is beneficial for energy expenditure, but increased circulating levels of BCAAs are detrimental to metabolism, so the mechanisms of these paradoxes need to be further explored.^159^ In recent years, Chaudhari et al have found that the altered gut microbiota (decreased Clostridia) following SG produced a microbial metabolism‐CA7S that increased plasma GLP‐1 level, thereby remodeling the gut‐liver axis to improve metabolism.^156^ They also showed that transferring of post‐SG microbiota to germ‐free mice recreated the CA7S pathway.^156^ In other words, changes in gut microbiota, Bas, and intestinal hormones resulting from alterations in gut anatomy and physiology connect the gut‐liver axis. Fecal microbiota transplantation (FMT) is generally performed in mice, and it has also been conducted in humans. Vrieze et al^177^ showed that insulin sensitivity increased 6 weeks after infusion of lean donor gut microbiota in male recipients with metabolic syndrome. Allegretti et al^178^ conducted a randomized trial to investigate the effects of FMT (derived from lean donors) in obese, metabolically uncompromised patients and showed that FMT did not reduce BMI in recipients but resulted in sustained changes in the gut microbiome and bile acid profile similar to lean donors. Recognized microbial changes following SG and RYGB include an increase in the relative abundance of Proteobacteria and a decrease in Firmicutes.^179^ An increase in Proteobacteria has also been reported after improvement in glucose homeostasis induced by metformin treatment, suggesting that Proteobacteria may be involved in metabolic improvement.^180^ Proteobacteria was increased in the fecal contents of mice in the SG group compared with the sham‐operated group.^156^ Firmicutes (dominant in obese individuals) and *Romboutsia* were significantly decreased in individuals after BS (such as GBP, RYGB, and SG), which is associated with significant weight loss, improved insulin resistance, and decreased systemic inflammation.^181^, ^182^, ^183^ In obese and T2D mice, the abundance of *Akkermansia muciniphila* in the gut is decreased, and its elevation after BS^44^ has been reported to reduce fat mass, improve metabolism in mice^184^, ^185^ and humans,^186^ increase thermogenesis by inducing UCP‐1 in BAT, and induce systemic GLP‐1 secretion in mice.^187^ Recently, Munzker et al have shown that depletion of the gut microbiota largely reversed the beneficial effects of GBP and intestinal microbiota after surgery regulated metabolism by reactivating thermogenesis in BAT through the FXR‐TGR5 crosstalk.^158^ Tremaroli et al found similar and durable gut microbiome changes in patients undergoing RYGB or VBG that were independent of body weight. Moreover, the surgically altered microbiome was demonstrated by FMT to promote the reduction in fat deposition, which further suggests that the gut microbiota may play a direct role in weight loss after BS.^188^ A recent study revealed that absolute deficiency of bacterial biotin producers and transporters was correlated with inflammatory phenotype and metabolic disorders in obese individuals. BS increased bacterial biotin producers to improve host systemic biotin in humans and mice, thereby improving metabolism and inflammation.^189^ A Chinese study found that probiotics + berberine (a natural bacteriostatic alkaloid derived from Berberis aristata and Huanglian) had better effects on lowering glycated hemoglobin in 409 participants with T2D. The result showed that berberine exerted glucose‐lowering effects through potential microbial mechanisms, further illustrating the critical role of gut microbiome in regulating host metabolism.^190^ In the future, combined management using gut‐centered therapies and B vitamins, including biotin, appears to be of interest in preventing the transition of obesity and T2D to a more severe metabolic state. Samczuk et al^191^ indicated that the recovery rate of T2D after SG may be related to the changes in gut microbiota composition and its effect on mitochondrial metabolism, and more investigations are still needed to explore these findings.

### 2.9 Changes of different brain function and food preferences

Obesity negatively affects brain function. Now, increasing studies have used fMRI to observe changes in brain activity in response to food cues in an effort to gain a deeper understanding about the mechanisms of BS. RYGB resulted in different brain responses compared with a very‐low‐calorie diets in clinical trials: RYGB resulted in a more active homeostatic appetite system, as well as reduced neural activation in response to food cues in cognitive control regions and responsiveness to food cues in the reward center of the brain,^192^ resulting in favorable changes in food rewards and preferences.^10^, ^193^ Another study, by utilizing functional brain imaging, reported that brain responses to high‐fat milkshake cues normalized at 1 year following RYGB in obese participants.^194^ In the fMRI study designed by De Silva et al, combined administration of PYY (3–36) and GLP‐1 (7–36 amide) to 15 fasted human subjects resulted in reduction in energy intake and brain activity.^195^ A study designed by Farr et al revealed that elevated GIP levels were associated with deactivation of insula related to attention and reward and decreased leptin levels were associated with activation or deactivation of different brain regions.^196^ GLP‐1R has also been demonstrated to be expressed in the hypothalamus, medulla oblongata, parietal cortex and so on.^197^ The effects of these gut hormones on the brain highlight the importance of the gut‐brain axis in controlling reward‐based eating behavior. The pro‐opio‐melanocortin (POMC) neurons and agouti‐related protein (AGRP) neurons located in the arcuate nucleus of the hypothalamus are the cores of gut‐brain axis, regulating blood glucose and metabolism through changes in their excitability. For instance, GLP‐2 increases the excitability of POMC neurons by activating GLP‐2R‐PI3K signaling, thereby reducing hepatic glucose production.^198^ However, existing studies are inconsistent with the changes of activity of POMC and AGRP neurons following BS by measuring the mRNA expression. In addition, the decrease in adipokines and inflammatory factors after BS may also be related to favorable changes in brain volume and cerebral blood flow.^199^, ^200^

Gustatory and olfactory function assessments in 68 participants undergoing RYGB or SG showed that BS may have positive effects on gustatory and olfactory function and eating behavior, with decreased hunger after surgery.^201^ However, the results from sensory studies are variable and limited and it has been showed that gustatory changes are not associated with the surgery‐mediated alterations in major intestinal appetite‐regulating hormones.^202^ Changes in food preferences and choices may contribute to the long‐term benefits of BS. Specifically, many studies have reported changes of food preferences following BS, including reductions in total fat and calorie intake and an increase in protein intake, and these changes were more common among participants who undergoing RYGB.^203^, ^204^, ^205^, ^206^, ^207^ Elevated intestinal hormones such as GLP‐1, PYY, and OXM have been suggested as possible mediators of the beneficial effects of RYGB on appetite and food preferences. In addition to the changes in intestinal hormones, changes in food preferences may also be related to postoperative changes in taste sensitivity, and conditioned avoidance and related changes in feeding motivation learning after RYGB may be candidate mediators.^208^ In addition, Smith et al found that the taste‐induced activation in the ventral tegmental area changed greatly after RYGB, suggesting that RYGB may more effectively reset the neural processing of reward stimulation in obese patients, thereby rescuing the blunted activation of the mesolimbic pathways,^209^ but found that this effect seemed to be temporary at 1 year of follow‐up.^210^ However, there are also studies reporting that RYGB and SG did not affect food preferences, much research remains to be explored in the future.^211^, ^212^

## 3 LIMITATIONS OF THE CURRENT STUDIES

As an invasive surgery, BS, coupled with potential side effects such as postoperative infection, anastomotic fistula, and malnutrition, cannot be widely applied to the population with surgical indications, and people are more inclined to choose pharmaceutical treatment. Therefore, most existing study populations on BS are limited, and future validation in larger populations is needed.

Most invasive research, such as studying changes in the composition of BAs in the portal vein after BS, has been conducted in animal models such as mice. We should be skeptical that whether the changes observed in animal studies are similar in humans. Although animal studies may provide ideas, the results should not be blindly extrapolated to humans without caution.

The study design, especially the control of confounding factors, should be determined before establishing the animal model of BS, so as to obtain convincing results. In addition, the involvement of other mechanisms should be considered comprehensively when studying one mechanism. For example, the secretion of intestinal hormones after BS is not only influenced by structural changes of the gastrointestinal tract but also by the brain. Therefore, future research should consider the comprehensive interaction among these mechanisms.

## 4 CONCLUSIONS

**FIGURE 1:**
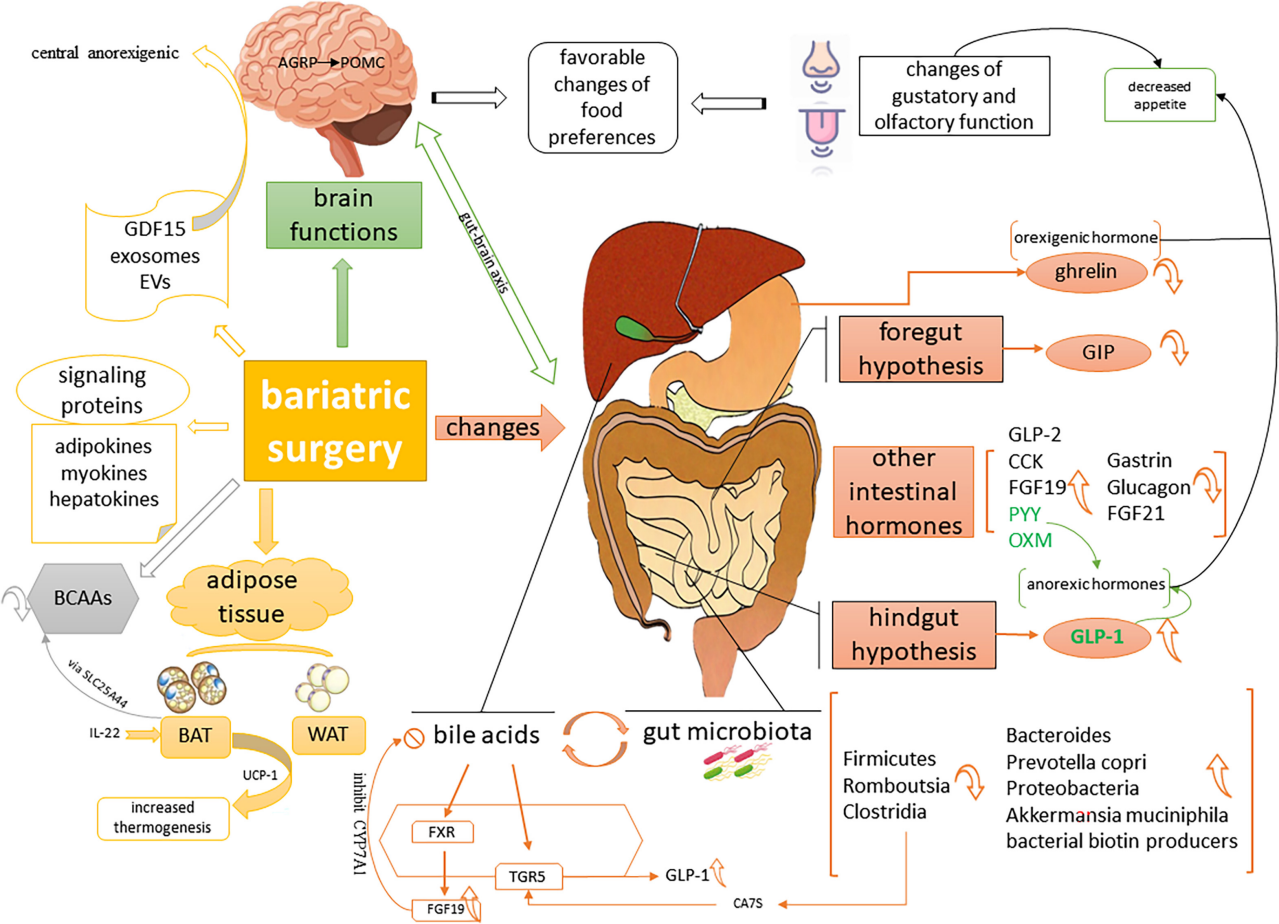
Main mechanisms associated with weight loss and remission of type 2 diabetes after bariatric surgery. The changes in gastrointestinal structure and function after bariatric surgery alter circulating hormones, gut microbiota, bile acids, thereby improving metabolism by ameliorating insulin resistance, reducing hepatic glucose output, and decreasing inflammation. In addition, the changes in adipose tissue and related factors after bariatric surgery also improve insulin resistance and reduce inflammation. At the same time, alterations in intestinal factors (through the gut‐brain axis), the central effects of certain factors such as GDF15, and the bariatric surgery itself directly or indirectly affect brain functions, thereby altering food selection, regulating appetite, and reducing energy intake. Together, these factors contribute to the remission of obesity and type 2 diabetes. AGRP, agouti‐related protein; BAT, brown adipose tissue; BCAAs, branched‐chain amino acids; CA7S, cholic acid‐7‐sulfate; CCK, cholecystokinin; CYP7A1, cholesterol 7a‐hydroxylase; EVs, extracellular vesicles; FGF‐19, fibroblast growth factor‐19; FGF‐21, fibroblast growth factor‐21; FXR, farnesoid X receptor; GDF15, growth differentiation factor 15; GIP, glucose‐dependent insulinotropic polypeptide; GLP‐1, glucagon‐like peptide‐1; GLP‐2, glucagon‐like peptide‐2; IL‐27, interleukin‐27; OXM, oxyntomodulin; POMC, pro‐opio‐melanocortin; PYY, peptide YY; SLC25A44, solute carrier family 25 member 44; TGR5, takeda G‐protein receptor 5; UCP‐1, uncoupling protein 1; WAT, white adipose tissue.

In this review, we comprehensively explore the multifactorial mechanisms underlying the beneficial effects of BS on weight loss and metabolism improvement (Figure 1). BS leads to weight loss, thereby reducing the fat content, changing the intestinal hormones, BAs, BCAAs, gut microbiota, signaling proteins (adipokines, myokines, hepatokines), GDF15, exosomes, brain function, and food preferences. However, as described in this review, none of these mechanisms appears to fully explain the beneficial effects of BS and different perspectives on the underlying mechanisms of BS remain to be elucidated. Further studies are needed to uncover the mechanisms behind BS, so as to provide new ideas for the treatment of obesity and metabolic disorders.

## AUTHOR CONTRIBUTIONS

Mengsha Yin wrote the manuscript. Yao Wang and Mingyue Han designed the illustrations. Ruishuang Liang and Shanshan Li helped to analyze literature. Guixia Wang and Xiaokun Gang edited and revised the manuscript. All authors have read and approved the final manuscript.

## CONFLICT OF INTEREST STATEMENT

The authors have no conflicts of interest to declare.
